# Optimization of lead compounds into on-demand, nonhormonal contraceptives: leveraging a public–private drug discovery institute collaboration^†^

**DOI:** 10.1093/biolre/ioaa052

**Published:** 2020-04-20

**Authors:** Melanie Balbach, Makoto Fushimi, David J Huggins, Clemens Steegborn, Peter T Meinke, Lonny R Levin, Jochen Buck

**Affiliations:** 1 Department of Pharmacology, Weill Cornell Medicine, New York, NY, USA; 2 Tri-Institutional Therapeutics Discovery Institute, New York, NY, USA; 3 Department of Physiology and Biophysics, Weill Cornell Medicine, New York, NY, USA; 4 Department of Biochemistry, University of Bayreuth, Bayreuth, Germany

**Keywords:** capacitation, soluble adenylyl cyclase, male contraception

## Abstract

Efforts to develop new male or female nonhormonal, orally available contraceptives assume that to be effective and safe, targets must be (1) essential for fertility; (2) amenable to targeting by small-molecule inhibitors; and (3) restricted to the germline. In this perspective, we question the third assumption and propose that despite its wide expression, soluble adenylyl cyclase (sAC: ADCY10), which is essential for male fertility, is a valid target. We hypothesize that an acute-acting sAC inhibitor may provide orally available, on-demand, nonhormonal contraception for men without adverse, mechanism-based effects. To test this concept, we describe a collaboration between academia and the unique capabilities of a public-private drug discovery institute.

## Teaser

An acute-acting inhibitor of soluble adenylyl cyclase (sAC: ADCY10) may provide orally available, on-demand, non-hormonal contraception for men without adverse, mechanism-based effects.

## A novel strategy for male contraception

With existing contraceptive options, preventing unintended pregnancies is largely the responsibility of females, for which several options exist. Female methods with greater than 99% success rates include tubal ligation, which is permanent, and intrauterine devices or hormonal implants, which require insertion by a doctor [1]. User-controlled barrier methods for females (i.e., diaphragms, sponges, or spermicides) result in failure rates greater than 13%. Finally, the only orally delivered methods available are hormone-based pills exclusively for women. Oral contraceptives require long-term use, carry significant side effects that are not easily tolerated by many women, and have failure rates up to 4–7%. In stark contrast, men have only two real choices: surgical vasectomy and condoms. Vasectomy has failure rates as low as 0.15% and is extremely effective, but it is largely irreversible [1] and therefore unsuitable for many men. On the other end of the spectrum, condoms supply on-demand contraception, but largely due to improper use, they have a typical failure rate of 13% and suffer from compliance issues; men (or couples) often report disliking their use due to discomfort or inconvenience [2]. Despite these drawbacks, condoms have been widely used since the time of the Roman Empire, which means that, except for surgery, male contraception has not meaningfully advanced for 2000 years [2, 3]. Thus, there is a profound need for new contraceptive strategies with an emphasis on nonhormonal methods and an even greater emphasis on methods enlisting males. Up till now, efforts to develop a male contraceptive focused exclusively on targets addressing three key questions: (i) Is it essential for spermatozoa development or function? (ii) Can it be blocked with specific and reversible pharmacological agents? and (iii) Is it exclusively functioning in the male germ cell? The final criteria were believed essential to ensure that the target could be safely blocked without any adverse, mechanism-based side effects. However, we now present a viable alternative: We propose a strategy where a fast-acting, reversible pharmacological agent against a target that satisfies only the first two criteria may be able to provide safe and effective, orally available, nonhormonal, and “on-demand” contraception for men.

## Soluble adenylyl cyclase is a unique enzyme essential for male fertility in mice and humans

Cyclic AMP (cAMP) is a nearly universally utilized second messenger molecule mediating signals throughout the bacterial and animal kingdoms. cAMP is synthesized by a broad family of adenylyl cyclases, and mammals possess two distinct classes of adenylyl cyclases: transmembrane adenylyl cyclases (tmACs) and soluble adenylyl cyclase (sAC) [4]. The tmACs are regulated by heterotrimeric G proteins and mediate cellular responses to intercellular signals, including hormones and neurotransmitters. For decades, the well-characterized family of tmACs (ADCY1—ADCY9) was thought to be the sole source of cAMP in mammalian cells. Prior to its molecular isolation [5], sAC was studied by following its biochemical activity. From these studies, soluble AC activity was predicted to be present only in testis [6]; specifically, it was postulated to be restricted to male germ cells. Its activity first appeared concomitantly with the development of spermatids in rats [7, 8] and humans [9], was missing in testicular feminized rats that contain little or no haploid germ cells [10], and was present in testis fractions enriched for spermatids [8, 10]. A biochemically related activity was detected in spermatozoa, and its activity was thought to be stimulated by sodium bicarbonate [11–14]. In 1999, we successfully purified and cloned sAC (ADCY10), defining a distinct adenylyl cyclase family in mammals [5].

**Figure 1:**
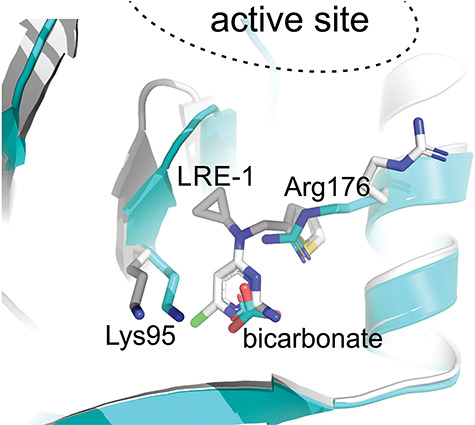
Crystal structures of ligands bound to sAC BBS. Crystal structure of bicarbonate in the sAC BBS (grey, PDB ID 4CLL), superimposed with the sAC − LRE1 complex (turquoise; PDB ID 5IV3). LRE1, bicarbonate, and the key bicarbonate-recognizing residues are shown in stick representation. The location of the active site where ATP binds is indicated [23, 46].

We purified a 50 kDa isoform of sAC from rat testis, which enabled isolating ADCY10 cDNAs encoding the full-length isoform of sAC (sAC_fl_) [5]. At its amino terminus, two related nucleotidyl cyclase catalytic domains form a generic class III AC catalytic core, which is necessary and sufficient for catalytic activity. Following the catalytic region is a long carboxy-terminus whose function remains largely unexplored. This carboxy terminus contains an autoinhibitory domain [15], a heme-binding domain [16], and based on weak sequence similarities, a putative STAND module [17]. However, how these presumptive regulatory domains modulate sAC activity remains unknown. Alternative splicing results in a premature stop codon [18] to generate a “truncated” sAC isoform (sAC_t_). sAC_t_ primarily consists of the two catalytic domains and corresponds to the ~ 50 kDa isoform we purified from testis. Heterologous expression of the cloned sAC transcripts [5, 18, 19] and purification of the heterologously expressed sAC_t_ [20, 21] protein clarified the biochemical distinctions between sAC and tmACs (reviewed in [4]). While insensitive to the known activators of tmACs, heterotrimeric G proteins [8] and forskolin [22], sAC activity is uniquely stimulated by bicarbonate, which accelerates substrate turnover [20, 21]. Crystal structures of the catalytic domain of human sAC and its complexes with substrates, products, bicarbonate, and analogs revealed the bicarbonate binding site (BBS) and identified local rearrangements contributing to activation (Figure 1) [23]. The sAC BBS is analogous to the forskolin binding site in tmACs, defining this as a general, regulatory site in mammalian adenylyl cyclases and providing a structural basis for the activator selectivity between sAC and tmACs. Forskolin, which is inert on sAC [5, 24], does not fit into sAC’s tighter, positively charged BBS [23], and bicarbonate does not bind to the wide, hydrophobic tmAC site lacking the bicarbonate recognizing residues. sAC is also regulated by calcium, which modulates the enzyme’s affinity for substrate ATP [19, 21], and its catalytic activity is sensitive to physiologically relevant changes in cellular ATP levels [21, 25].

In sperm, sAC is the major cAMP-generating enzyme, crucial for sperm motility and capacitation (reviewed in [26, 27]). Capacitation is the essential maturation process required for sperm to acquire fertilization competence; it commences upon ejaculation and continues as sperm transit through the female tract [28, 29]. Upon leaving the testes, mammalian sperms are morphologically mature, but unable to fertilize an oocyte. They are stored in the cauda region of the epididymis in an environment characterized by a low pH (i.e., 6.5–6.8 instead of 7.4) and low HCO_3_^−^ concentration (i.e., 2–7 mM instead of 25 mM) [30]. This unique epididymal luminal environment maintains the sperm in a dormant state. Upon ejaculation, sperms come into contact with seminal fluid; its high HCO_3_^−^ and Ca^2+^ concentrations [31, 32] synergize to activate sAC [19, 21, 33, 34]. The activation of sAC rapidly (i.e., within seconds) elevates sperm cAMP, which increases the flagellar beat frequency more than 2-fold [35]. Two independently generated strains of mice with ADCY10 knocked out (KO) exhibit male-specific sterility [35–37]; sAC-deficient sperms lack cAMP synthesis, are immotile, and do not display molecular hallmarks normally accompanying capacitation [37, 38]. Recently, this phenotype was identified in humans. In 2019, two infertile male patients were reported who were homozygous for a frameshift mutation in the exonic region of *ADCY10*, leading to premature termination and interruption of the catalytic domains [39]. Similar to sAC null mice, sperm from those patients are immotile, and this motility defect could be rescued with cell-permeable cAMP analogs. Thus, sAC satisfies the first criteria as a potential target for a male contraceptive: It is essential in sperm for male fertility in mice and men.

**Figure 2:**
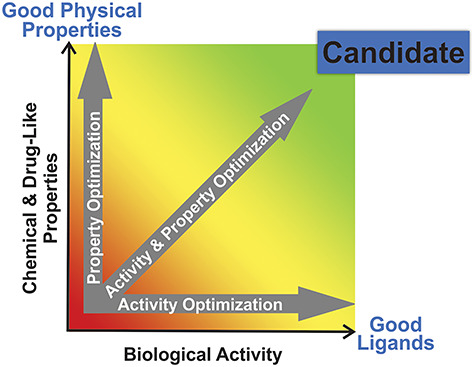
Modulating sAC inhibitors to discover a preclinical candidate. Molecular property optimization directed toward preclinical candidate selection; final candidate (dark blue) is the analog that best balances overall desirable properties versus ligand-associated liabilities.

**Figure 3:**
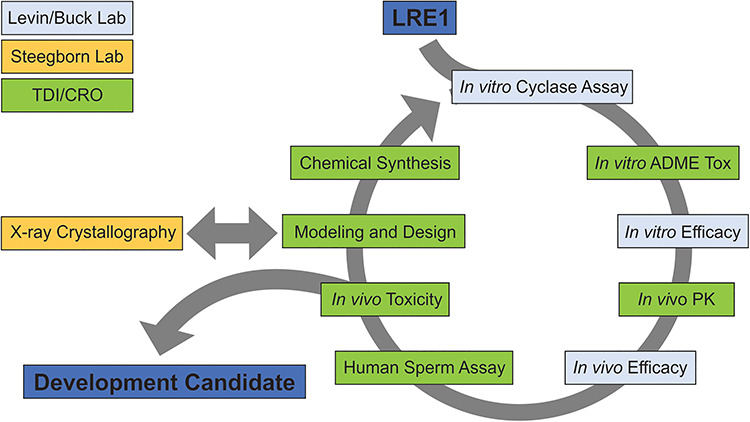
The iterative cycle for designing, synthesizing, and evaluating sAC inhibitors. Starting from LRE1, the workflow used to identify a preclinical development candidate includes activities performed in the Levin/Buck laboratory (light blue), the Steegborn laboratory (orange), or at TDI or TDI-managed CROs (green). *In vitro* efficacy models include both cell-based cyclase assay and *in vitro* efficacy mouse sperm. Structural biology information is used to model and design new compounds.

## sAC can be selectively and reversibly inhibited by small molecules

Following our molecular identification of sAC, to be able to spatially and temporally probe its functions, we required ligands that modulate sAC without affecting tmACs The first known sAC inhibitors were catechol estrogens (CE; see Table 1 for a summary of sAC inhibitors), which were found to inhibit noncompetitively through binding to a groove near the active site and chelating a divalent cation essential for adenylyl cyclase activity [40]. While CEs demonstrated an ability to selectively inhibit sAC in cellular systems [41, 42], they are not specific for sAC relative to tmACs [40]. To satisfy our need for sAC-selective pharmacological tools, we identified the sAC-specific inhibitor KH7 in a small molecule high throughput screen (HTS) [37]. KH7 is inert against tmACs and cell-permeable and inhibits sAC in tissues and animals [37, 43]. KH7 has grown into the most widely used pharmacological agent for identifying sAC functions [44], including blocking sperm capacitation and in vitro fertilization (IVF) [37]. Despite its widespread use, KH7 has liabilities that result in sAC-independent toxicity [45]. In a subsequent HTS using human sAC, we identified the chemically distinct, sAC-specific inhibitor LRE1 [46]; LRE1 also blocks the sAC-mediated functions in sperm. Thus, two structurally distinct inhibitors block sAC-dependent functions in sperm essential for fertilization, confirming the second criteria for developing a male contraceptive; sAC is amenable to targeting by small-molecule inhibitors.

**Table 1:** Chemical structures of sAC inhibitors described in the text along with the PDB reference for their three-dimensional structure complexed with sAC (where available).

Name	Chemical structure	sAC complex structure (PDB entry)	Reference
CE (2HE)	FX1	2BW7 (bacterial sAC homolog CyaC)	[40]
KH7	FX2	Not available	[37]
DIDS	FX3	4CLZ	[23]
ASI-8	FX4	4OYA	[59]
Bithionol	FX5	5D0R	[60]
LRE1	FX6	5IV3 5IV4	[46]

### sAC is widely expressed

Soon after our molecular isolation of sAC, it became clear that the third criteria for a male contraceptive posed significant challenges. Up to that point, biochemical characterization of soluble adenylyl cyclase activity suggested that sAC expression was restricted to male germ cells, and initial Northern blot, RT-PCR, and in situ hybridization experiments confirmed that sAC expression was indeed highest in male germ cells [5, 47]. However, these and other studies [20, 48, 49] revealed that sAC is also widely expressed, albeit at low levels. And consistent with widespread distribution, genetic and pharmacological experiments identified roles for sAC in a number of physiological processes in addition to male fertility (reviewed in [44, 50–52]). For example, sAC in somatic tissues mediates the cAMP-dependent signaling cascades that regulate luminal pH in the epididymis [42]; ciliary beat frequency in airway epithelia in response to elevated CO_2_ [53, 54]; regulation of intraocular pressure [43, 55]; and leukocyte migration [56].

These somatic functions were assumed to complicate sAC‘s contraceptive potential. However, the two infertile male patients homozygous for inactivating mutations in sAC are healthy adults; besides infertility, their only reported health issue is increased incidence of kidney stones [39]. Similarly, the sole overt phenotype in the two molecularly distinct sAC KO mouse strains is male-specific sterility [35–37]. Other phenotypes observed in sAC KO mice (reviewed in [44]) and men [39] are conditional (i.e., decreased airway ciliary beat frequency in response to elevated CO_2_), or they are not expected to be detrimental when transiently induced (i.e., increased risk of kidney stones, increased intraocular pressure, and decreased leukocyte migration). Thus, although sAC is widely expressed, the effects of its loss are primarily restricted to male infertility, and it appears that somatic functions of sAC-generated cAMP are likely to be tolerated if sAC function is acutely inhibited.

We also considered the example of another widely expressed gene that remains safe even when systemically targeted. PDE5 is expressed in multiple tissues [57], yet sildenafil, vardenafil and tadalafil, which are acute PDE5 inhibitors (half-lives 4–17.5 h), are sufficiently safe for treating erectile dysfunction. These PDE5 inhibitors teach us that acute inhibition can be markedly different from chronic loss. Thus, we propose that by carefully controlling the time and dose of a fast-acting, reversible sAC inhibitor, acute administration can provide on-demand, reversible, effective contraception without adverse, mechanism-based effects.

## Rational design acute inhibitors of sAC to provide on-demand contraception

TmACs are the enzymes most closely related to sAC in mammalian genomes; thus, selective sAC-inhibitors must be inert against tmACs. Active site differences between sAC and tmACs are subtle, making it an improbable site for selective inhibitors. In contrast, because only sAC is regulated by bicarbonate [20, 21, 23, 58], sAC’s allosteric BBS has potential as a site for sAC-specific inhibitors. A first compound studied for exploiting the sAC-specific BBS was 4,4′-diisothiocyanatostilbene-2,2′-disulfonic acid (DIDS), a bicarbonate transporter blocker that was speculated to enter the BBS with one of its sulfonic acid moieties. A sAC complex structure revealed, however, that it binds at the active site entrance, blocking access to the active site and BBS [23]. Thus far, three small molecules were structurally identified to occupy the BBS: (1) ASI-8 occupies the BBS and extends into the active site [59]; (2) the organochloride bithionol occupies the mostly hydrophobic BBS access channel for a mixed-type inhibition with respect to ATP and positions a chlorine in the bicarbonate pocket [60]; and (3) LRE1. Crystal structures of sAC/LRE1 complexes revealed that the compound’s 2-amino-6-chloropyrimidine occupies the BBS and its small cyclopropyl moiety reaches into the channel connecting BBS and active site but does not overlap with ATP binding regions (Figure 1) [46]. Consistently, inhibition by LRE1 was found to be competitive with bicarbonate but noncompetitive with substrate, defining it as the first fully allosteric BBS-targeting sAC inhibitor. Although LRE1 is a nontoxic, sAC-selective inhibitor that prevented sAC functions in sperm, it is precluded from development due to its myriad flaws. LRE1 deficiencies include modest intrinsic potency, negligible cellular activity, poor pharmacokinetic characteristics (including poor bioavailability < 5%, high intrinsic clearance, and high metabolic rate), plus its thiophene moiety poses significant safety concerns (FDA structural alert for reactive metabolites [58]). Importantly, the apo- and ligand-bound sAC structures provide unique insights into the precise mode of binding and key contacts between LRE1 and sAC (Figure 1) [23, 46]. As a result, despite its glaring defects, LRE1 represents an auspicious starting point for medicinal chemistry efforts.

The expertise required to rectify the deficiencies in LRE1 to develop an acute, drug-like sAC inhibitor historically has been in the purview of the pharmaceutical industry. Unfortunately, attracting the interest of pharma is problematic for any novel target, and it is even more challenging for an idea as nascent as an acute inhibitor providing on-demand male contraception. With the expectation that pharma is too risk-averse to address such an innovative and provocative theory, where can an academic laboratory turn for drug discovery assistance?

## TDI provides a novel pathway to discover new drugs

In 2013, Weill Cornell Medicine formed a unique public-private partnership with Memorial Sloan Kettering Cancer Center and the Rockefeller University to launch the Tri-Institutional Therapeutics Discovery Institute, Inc. (TDI). TDI is a nonprofit [501(c)(3)] organization made possible through funding from the three founding institutions, an industry partner, Takeda Pharmaceutical Company, Ltd. (Takeda), and philanthropic donors. TDI links academic researchers in the biomedical sciences with industry experts in drug discovery to more efficiently translate groundbreaking discoveries into clinical applications.

The mission of TDI is to accelerate academic drug discovery. Since its inception, TDI has evolved best practices for identifying and executing academic-initiated drug discovery projects. TDI invests considerable effort in promising drug discovery projects from across the three participating institutions, such as the sAC inhibitor program, which was invited to apply for TDI’s Therapeutic Initiative. Each proposal is subjected to rigorous review by its independent external Scientific Advisory Board, comprised of recognized experts across the industry. TDI is both therapeutic area agnostic and modality independent, operating both small molecule and biologics discovery teams. Projects are selected solely based on scientific merit and unmet medical need.

Thus far, since its inception, TDI contributed to the launch of two NYC-based companies and the licensing of seven additional institutional assets (five small molecules, two biologics), with 4 more licensing agreements under negotiation (one small molecule and three biologics). In addition, TDI tools and reagents have substantially strengthened grant applications and publications, as well as creating entirely novel research opportunities, using these materials to more deeply interrogate biological targets.

TDI’s internal expertise in medicinal chemistry, computational chemistry, pharmacology, immunology, biologics, and project management advances projects from academic discovery to viable Investigational New Drug (IND) candidates. TDI maintains an extensive network of contract research organizations (CROs, currently > 100) and expert consultants to address project-specific needs that cannot be met internally.

At the conclusion of a therapeutic program, TDI delivers a comprehensive graduation document for each product that achieves successful proof-of-concept in a disease-relevant, in vivo model. Throughout this process, TDI works closely with the PI’s parent institution, which controls all intellectual property rights on the project, to assist in commercialization activities. It should be noted that the originating institution is not obligated to partner with Takeda and retains optionality for selecting alternative development pathways.

## Strategy for refining the existing LRE1 scaffold into an acute drug-like molecule

The goal of this sAC inhibitor project is to refine the LRE1 scaffold to maximize biological efficacy and drug-like physical properties to identify an acute sAC inhibitor that merits the considerable resource investment as a preclinical development candidate (Figure 2) and the subsequent accumulation of the requisite data package essential to support an Investigational New Drug (IND) application to the FDA. Specific steps in this discovery process are conceptually illustrated in the sAC inhibitor program workflow (Figure 3). Structural biology data, married to computational support, allows TDI medicinal chemists to iteratively design and dock potential new ligands into the BBS prior to their synthesis. Moreover, ligand optimization advances through the program by subsequently engineering in enhanced “drug-like” properties to permit their ready absorption as orally dosed agents, minimize metabolic and half-life issues, and build in target specificity while reducing activities at undesired receptors. Synthesized potential inhibitors are tested on human sAC protein in vitro cyclase assays to determine their potency. This iterative design/synthesize/test process greatly accelerates the ligand optimization process to efficiently identify ligands with appropriate intrinsic potency for the sAC binding site.

sAC inhibitors with improved potencies are screened against tmACs for selectivity and tested in ancillary assays for safety. TDI exploits its existing CRO network for absorption, distribution, metabolism, excretion, and toxicity (ADME-Tox) studies and pharmacokinetics (PK). Ideal candidates for further development will be sAC-specific inhibitors which are safe, have no off-target effects and are orally bioavailable with rapid binding to sperm stored within the epididymis and/or prostate fluid. It remains to be established whether fast-acting, but high clearance compounds constitute an “ideal drug profile,” or whether chronically administered agents with a longer duration of action might prove to be superior. Therefore, pursuing multiple candidates with a range of PK profiles will maximize the likelihood for identifying a dosing regimen that optimally balances the onset of action with duration of efficacy and mechanism-based safety characteristics for this contraceptive mechanism. Inhibitors with appropriate ADME-Tox and PK profiles are injected into animals to test for blockage of sperm capacitation and fertilization. The ultimate goal is to identify a sAC inhibitor that provides on-demand contraception within an hour after oral dosing. The unique aspect of an acute on-demand pharmacological agent allows effective contraception, while avoiding the potential side effects of chronic, long-term sAC inhibition. For example, the ideal sAC inhibitor male contraceptive will block sperm functions for sufficient time to afford convenience without inducing putative mechanism-based side effects, such as kidney stones [39].

By leveraging the unique capabilities of a public-private drug discovery institute, TDI, in collaboration with academic laboratories, we have made considerable improvements in the LRE1 scaffold. Novel sAC inhibitors with improved potency and drug-like characteristics [manuscript in preparation] will allow testing the innovative and potentially revolutionary strategy providing orally available, on-demand, nonhormonal contraception for men.

## Author contributions

M.B., M.F., D.J.H., P.T.M., C.S., L.R.L., and J.B. conceived of the ideas and wrote the manuscript.

## Conflict of interest

Drs. Buck and Levin own equity interest in CEP Biotech, which has licensed commercialization of a panel of monoclonal antibodies directed against sAC. All other authors declare that they have no conflicts of interest with the contents of this article.
